# Genetic Variability of Hepatitis C Virus (HCV) 5’ Untranslated Region in HIV/HCV Coinfected Patients Treated with Pegylated Interferon and Ribavirin

**DOI:** 10.1371/journal.pone.0125604

**Published:** 2015-05-01

**Authors:** Iwona Bukowska-Ośko, Agnieszka Pawełczyk, Karol Perlejewski, Natalia Kubisa, Kamila Caraballo Cortés, Magdalena Rosińska, Rafał Płoski, Maria Fic, Justyna Kaźmierczak, Marta Popiel, Piotr Ząbek, Andrzej Horban, Marek Radkowski, Tomasz Laskus

**Affiliations:** 1 Department of Immunopathology, Warsaw Medical University, Warsaw, Poland; 2 Department of Epidemiology, National Institute of Hygiene, Warsaw, Poland; 3 Department of the Medical Genetics, Warsaw Medical University, Warsaw, Poland; 4 Hospital of Infectious Diseases, Warsaw, Poland; National Taiwan University Hospital, TAIWAN

## Abstract

Association between hepatitis C virus (HCV) quasispecies and treatment outcome among patients with chronic hepatitis C has been the subject of many studies. However, these studies focused mainly on viral variable regions (E1 and E2) and usually did not include human immunodeficiency virus (HIV)-positive patients. The aim of the present study was to analyze heterogeneity of the 5'untranslated region (5'UTR) in HCV/HIV coinfected patients treated with interferon and ribavirin. The HCV 5’UTR was amplified from serum and peripheral blood mononuclear cells (PBMC) samples in 37 HCV/HIV coinfected patients treated for chronic hepatitis C. Samples were collected right before treatment, and at 2, 4, 6, 8, 12, 20, 24, 36, 44, 48, 60, and 72 weeks. Heterogeneity of the 5'UTR was analyzed by single strand conformational polymorphism (SSCP), cloning and sequencing. Sustained virological response (SVR) was achieved in 46% of analyzed HCV/HIV co-infected patients. Stable SSCP band pattern was observed in 22 patients (62.9%) and SVR rate among these patients was 23%. Decline in the number of bands and/or shift in band positions were found in 6 patients (17.1%), 5 (83%) of whom achieved SVR (p=0.009). A novel viral genotype was identified in all but one of these patients. In 5 of these 6 patients a new genotype was dominant. 5'UTR heterogeneity may correlate with interferon and ribavirin treatment outcome. In the analyzed group of HCV/HIV coinfected patients, viral quasispecies stability during treatment favored viral persistence, whereas decrease in the number of variants and/or emergence of new variants was associated with SVR. Among injection drug users (IDU) patients, a new genotype may become dominant during treatment, probably due to the presence of mixed infections with various strains, which have different susceptibility to treatment.

## Introduction

Due to similar routes of transmission, coinfection with hepatitis C virus (HCV) and human immunodeficiency virus (HIV) is common [1, 2]. Approximately 10–15% of the 35 million HIV-infected individuals worldwide are coinfected with HCV, while among 150 million of humans infected with HCV, the HIV coinfection rate is close to 3%. Altogether, roughly 4 million people are believed to be infected by both pathogens [3].

Patients with dual HCV/HIV infection are at a higher risk of developing decompensated liver disease and hepatocellular carcinoma [4–6]. As a consequence of introduction of highly active antiretroviral therapy (HAART) and lowering the overall HIV-related death rate, liver disease became the leading cause of death among HIV-positive patients [7]. Despite the somewhat lower rate of treatment success, it is widely accepted that HCV/HIV coinfected patients are likely to benefit from treatment regimens aimed at HCV eradication [8].

Similar to many other RNA viruses, HCV circulates as a heterogeneous population of closely-related variants, referred to as quasispecies [9–13]. This highly dynamic nature of quasispecies is believed to have major biological implications; thus variants differing in the E2 region, which codes major viral epitopes, could avoid immune neutralization, while 5' untranslated region (5' UTR) variants are likely to express different translation efficiency [14]. High rate of mutation could be responsible for adaptation properties of RNA viruses and could hamper the development of successful therapies [14]

Analysis of HCV quasispecies diversity in patients undergoing antiviral treatment has been the subject of many studies, but these were almost exclusively confined to viral variable regions like E1 and E2, and usually did not include HIV-positive patients [15–19].

The aim of the present study was to analyze heterogeneity of the 5'UTR in HCV/HIV coinfected patients treated with interferon and ribavirin.

## Patients and Methods

### Patients

The study group consisted of 37 HCV/HIV coinfected patients treated for chronic hepatitis C between June 2005 and July 2007 at the Municipal Hospital for Infectious Diseases, Warsaw, Poland.

There were 16 (43%) women and 21 (57%) men, their median age was 36 years (range 24–58). The predominant HCV genotypes were 1b and 4c/4d found in 12 patients each (32.5%), followed by 3a, which was present in 10 patients (27%). Median pretreatment HCV viral load was 522,400 IU per ml (interquartile range 721,500–2,145,000). Median CD4+ cell count was 516,74 per ml (range 303–1160), while median serum HIV load was 3,300 IU per ml (range 0–24,000). (Table 1) Twenty-one patients were on HAART at the time of the study and all were on methadone. Twenty-eight patients had history of injection drug use (IDU) but remained abstinent from one to 14 years prior to the initiation of treatment.

**Table 1:** Baseline characteristic of 37 HCV/HIV coinfected patients undergoing therapy with interferon and ribavirin.

Age	mean ±SD, years	36 ± 8.26
**Sex**	Female	16/37 (43.24%)
	Male	21/37 (56.76%)
**Genotype**	1b	12/37 (32.5%)
	3	10/37 (27%)
	4c/4d	12/37 (32.5%)
	undetermined	3/37 (8.1%)
**Starting HCV load, IU/ml (x 10^4)**	mean	52.24
**Starting HIV load, IU/ml (x 10^4)**	mean	0.33
**Putative infection route**	IDU^a^	28/37 (75.68%)
	MSM^b^	1/37 (2.7%)
	Unknown	8/37 (21.62%)
**CD4 cell count,** IU/ml	mean±SD	516.74 ± 194.48
**IL28 genotype**	C/C	15/37 (40.54%)
	T/T	5/37 (13.51%)
	C/T	13/37 (35.14%)
	no information	4/37 (10.81%)
**On HAART**		21/37 (56.76%)

^a^ IDU, injection drug users.

^b^MSM, men who have sex with men.

All patients were treated with peginterferon alfa-2a 180 μg/week (Pegasys; Hoffmann-LaRoche, Basel, Switzerland), or peginterferon alfa-2b 1.5 μg/kg/weekly (PegIntron; Schering-Plough Corp, Nenilworth, NI) combined with ribavirin (Schering-Plough Corp) in the dose of 1000 mg/day for patients weighing <75 kg or 1200 mg/day for patients weighing ≥75 kg. Patients infected with HCV genotype 3 were treated for 24 weeks, however, in those remaining HCV RNA positive at 24 weeks the therapy was extended to 48 weeks. Patients harbouring genotypes 1 or 4 were treated for 48 weeks. Sustained virological response (SVR), defined as negative HCV RNA 24 weeks after the end of treatment, was used as a measure of treatment outcome. Per current recommendations, the therapy was stopped in patients with HCV RNA load reduction <2 log_10_ IU/ml at week 12.

Presence of HCV RNA in serum was determined right before commencement of therapy (week 0) and 24 weeks after the end of therapy by a qualitative assay (COBAS AMPLICOR HCV Monitor Test, Roche Diagnostics, CA; detection limit of 50 IU per mL). Serum HCV RNA and HIV RNA viral load was quantified by ABBOTT Real Time PCR HCV CE and by ABBOTT Real Time PCR HIV CE (Abbott Molecular Inc., Des Plaines, IL), respectively, before treatment. In addition, the HCV load was quantified at 12 weeks.

HCV genotyping was performed using the INNO-LiPA assay (Innogenetics, NV, Gent, Belgium). The IL28B polymorphism (rs12979860) was analyzed using the TaqMan SNP genotyping assay (Applied Biosystems Inc, Foster City, CA, USA). Genotyping calls were verified with SDS software (Applied Biosystems Inc.).

Serum and PBMC samples were collected for analysis at the following time points: right before treatment, and at 2, 4, 6, 8, 12, 20, 24, 36, 44, 48, 60, and 72 weeks (24 weeks after the end of treatment).

### Laboratory methods

HCV 5’UTR was amplified by RT-PCR as described previously [20]. Sequence comparative analysis of purified DNA PCR products was performed by single strand conformational polymorphism (SSCP) method [21].

When SSCP analysis suggested the presence of sequence differences, samples were reamplified, cloned and sequenced. Cloning was performed using the TOPO TA Cloning Kit (Invitrogen). Isolated plasmids were sequenced bidirectionally using ABI 3130 Genetic Analyzer and BigDye Terminator v3.1 Kit (Applied Biosystem, Foster City, CA, USA). Six to 12 clones were analyzed for each cloned sample. Sequence alignments were done using MEGA program [22].

### Statistical analysis

Chi-square test was used to compare proportions and Mantel-Haenszel test was employed to identify difference in the impact of predictors on SVR. Due to limited sample size multivariate analysis was not feasible. A p-value <0.05 was considered statistically significant.

### Ethics statements

The study was approved by the Internal Review Board at the Warsaw Medical University (ref No KBO/23/09). Each patient signed an informed consent form.

## Results

Seventeen (46%) out of 37 patients achieved SVR and 20 (54%) were considered non-responders. The SVR rate in patients infected with HCV genotype 3a was 70%, whereas among patients infected with genotypes 1 or 4 only 37.5% cleared infection (Table 2).

**Table 2:** Univariate analysis of characteristics associated with sustained virological response (SVR) among 37 HCV/HIV coinfected patients receiving antiviral treatment.

Factor		N (%)	N SVR	Rate of SVR	RR (95% CI)	p-value
**TOTAL**		37	17	0.46		
sex	male	21 (56.8%)	7	0.33	ref.	0.078
	female	16 (43.2%)	10	0.63	1.88 (0.92–3.83)	
age	<30	10 (27%)	7	0.70	ref.	0.063
	30–49	24 (64.9%)	9	0.38	0.54 (0.28–1.03)	
	> = 50	1 (2.7%)	0	0.00	unestimable	
	missing	2 (5.4%)				
IL28	C/T	13 (35.1%)	6	0.46	2.31 (0.36–14.66)	0.431
	C/C	15 (40.5%)	8	0.53	2.67 (0.43–16.39)	
	T/T	5 (13.5%)	1	0.20	ref.	
	missing	4 (10.8%)				
genotype	other than 3a	24 (64.9%)	9	0.38	ref.	0.0836
	3a	10 (27%)	7	0.70	1.87 (0.97–3.6)	
	missing	3 (8.1%)				
starting HCV viral load (IU/ml)	<10^6^	22 (59.5%)	11	0.50	ref.	0.6418
	≥10^6^	12 (32.4%)	5	0.42	0.83 (0.38–1.83)	
	missing	3 (8.1%)				
HIV viral load (IU/ml)	undetectable	19 (51.4%)	7	0.37	ref.	0.198
	<10^4^	14 (37.8%)	9	0.64	1.74 (0.86–3.54)	
	≥10^4^	4 (10.8%)	1	0.25	0.68 (0.11–4.09)	
CD4	<500	20 (54.1%)	10	0.50	ref.	0.8085
	> = 500	11 (29.7%)	5	0.45	0.91 (0.42–1.99)	
	missing	6 (16.2%)				
antiretroviral therapy	no	16 (43.2%)	10	0.63	ref.	0.0778
	yes	21 (56.8%)	7	0.33	0.53 (0.26–1.09)	
shift	no	28 (75.7%)	10	0.36	ref.	0.033
	yes	6 (16.2%)	5	0.83	2.3 (1.26–4.30)	
type of SSCP changes^a^	stable	22 (62.9%)	5	0.23	ref.	0.009
	decline in no. of bands	6 (17.1%)	5	0.83	3.67 (1.57–8.57)	
	shift	6 (17.1%)	5	0.83	3.67 (1.57–8.57)	
	undetermined	1 (2.9%)				

^a^ Patients with at least one HCV RNA-positive serum or PBMC sample after treatment initiation; N = 35

No significant association was found between SVR and sex, age, IL28B genotype, baseline HCV and HIV viral load, HCV genotype, CD4+ cell count and HAART in univariate analysis, although some positive trends were noted with respect to female gender (p = 0.078), age under 30 (p = 0.063), HCV genotype 3a (0.083) and no HAART treatment (p = 0.078); (Table 2).

**Fig 1:**
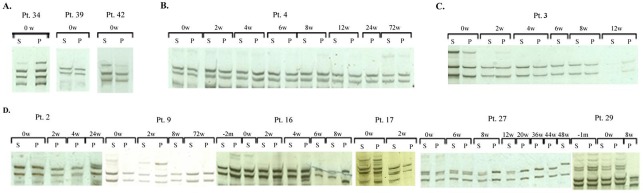
Different patterns of single-strand conformation polymorphism (SSCP) analysis of 5’untranslated region. A, comparison of SSCP band patterns in serum and PBMC before treatment; B, no changes in SSCP band pattern during treatment; C, decline in the number of SSCP bands without a shift in band positions; D, ‘shift’ in SSCP band pattern indicative of sequence changes. HCV RNA was amplified from serum (s) and PBMC (p) at numerous time-points (see the Methods section), but only positive samples are shown; w, weeks.

The pretreatment SSCP band patterns for serum and PBMC displayed no differences suggesting presence of identical viral population in both compartments in all analyzed patients. Examples of SSCP analysis of pretreatment serum and PBMC samples are shown in Fig 1A.

**Fig 2:**
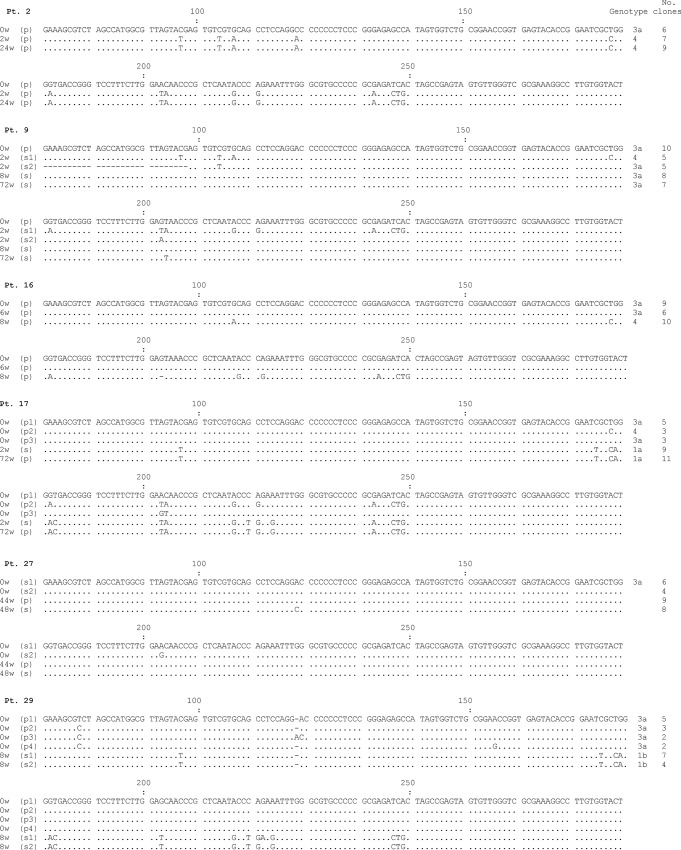
Multiple sequences alignment of 5’UTR variants detected in serum (s) and PBMC (p) of six patients with chronic hepatitis C in whom changing SSCP band pattern (shift) was suggestive of sequence changes. Dots indicate consensus positions. Six to 12 clones were analyzed for each particular time point and compartment. Only clones with different sequences are shown and the number of clones representing each sequences is provided in the Figure.

Three types of SSCP patterns were identified: (1) no changes in SSCP band pattern implying stability of viral quasispecies population (Fig 1B); (2) decrease in the number of SSCP bands in consecutive samples without positional changes implying decline in viral population complexity (Fig 1C); appearance of changes in SSCP band localization/position defined as ‘shift’ (Fig 1D).

Stable SSCP band pattern was the most common as it was observed in 22 patients (62.9%); (Table 2). Only 5 (23%) patients in this group achieved SVR and in all these patients SSCP band pattern remained stable until viral clearance. The other two SSCP band patterns were less common as each was found in 6 patients (17.1%). However, 5 (83%) out of 6 patients in each of these groups achieved SVR and this difference was statistically significant (p = 0.009).

The decrease in the number of SSCP bands, which was observed in 6 patients, occurred within the first 8 weeks of treatment. Similarly, a shift was observed within the same time span in 4 out of 6 patients. Among 6 patients in the shift group, in two (pt 9 and 17) the changes occurred simultaneously in serum and PBMC, while in another three patients (pt 2, 16 and 29) the shift was confined to the PBMC compartment (Fig 1D). Moreover, in two of the latter patients (pt 2 and 29) all serum follow up samples were HCV RNA negative. Thus, without analyzing of PBMC compartment, 3 out of 6 shifts would have been missed.

One 'shift' patient (pt 17) was HCV RNA positive for only 2 weeks, and two other patients (pt 16 and pt 29) were positive for 8 weeks. While patient 2 was positive until the 24 week sample, HCV RNA was present exclusively in PBMC, as the first serum follow up sample drawn at 2 weeks was already negative. HCV RNA in PBMC is not routinely tested during treatment and it has been reported that the virus could persist in this compartment longer than in serum [23, 24]. In patient 9 the last HCV RNA positive sample was drawn at 8 weeks, however, once the therapy was stopped he became again positive and he was deemed a non-responder. Interestingly, patient 27 remained positive until the end of therapy, but was negative 6 months thereafter and was considered therefore to be a responder. Such delayed viral clearance could have been be related to the concomitant HIV-infection.

The relationship between treatment outcome and various clinical, demographic and virological characteristics was analyzed separately in patients with stable and changing SSCP patterns (Table 3). Among patients with stable SSCP band pattern, the SVR was associated with female sex (p = 0.02) and no HAART (p = 0.04), while among patients with changing band pattern, who had an overall success rate of 83%, none of the analyzed factors was predictive of outcome. Viral genotype, IL28 polymorphism, clinical outcome and SSCP band patterns for each of the analyzed patients are shown in Table 4.

**Table 3:** Univariate analysis of characteristics associated with sustained virological response (SVR) among HCV/HIV coinfected patients with stable and changing SSCP patterns.

		Stable pattern					Changing pattern^a^					p-value for homogeneity of strata
		N	N SVR	Rate of SVR	RR (95% CI)	p-value	N	N SVR	Rate of SVR	RR (95% CI)	p-value	
**TOTAL**		22	5	.23			12	10	.83			
sex	male	14	1	.07	ref.	0.0210	6	5	.83	ref.	1.000	0.0101
	female	8	4	.5	7(0.94–52.34)		6	5	.83	1(0.6–1.66)		
age	<30	5	2	.40	ref.	0.2944	4	4	1	ref.	0.4279	0.1479
	> = 30	17	3	.18	0.44(0.1–1.95)		7	6	.86	0.86(0.63–1.16)		
IL28	CC or CT	14	2	.14	ref.	0.7636	12	10	.83	ref.	-	-
	TT	5	1	.2	1.4(0.16–12.29)		0	-	-	-		
genotype	other then 3a	16	3	.19	ref.	0.3302	6	5	.83	ref.	0.3384	0.2815
	3a	5	2	.40	2.13(0.49–9.38)		5	5	1	1.2(0.84–1.72)		
starting HCV viral load	<10^6^	14	4	.29	ref.	0.4687	6	6	1	ref.	0.2506	0.4995
	> = 10^6^	7	1	.14	0.5(0.07–3.67)		5	4	.80	0.8(0.52–2.72)		
antiretroviral therapy	no	9	4	.44	ref.	0.0431	6	5	.83	ref.	1.0000	0.020
	yes	13	1	.08	0.17(0.02–1.3)		6	5	.83	1(0.6–1.66)		

^a^ Decline in the number of bands or shift in band positions

**Table 4:** Viral genotype, IL28 polymorphism, clinical outcome and SSCP band pattern changes in 37 HCV/HIV coinfected patients undergoing antiviral treatment.

Type of SSCP band pattern change	Patient ID	IL28 genotype	HCV genotype	SVR
shift	2	C/C	3a	yes
	9	C/C	undetermined	no
	16	C/T	3a	yes
	17	C/T	3a	yes
	27	C/T	3a	yes
	29	C/T	3a	yes
decrease	3	C/C	1b	yes
	22	C/C	4c/4d	yes
	32	C/C	4c/4d	yes
	41	C/T	1b	yes
	50	C/C	1b	yes
	167	C/C	1b	no
stable	4	C/T	1b	no
	8	C/T	4c/4d	no
	11	T/T	4c/4d	no
	13	C/C	1b	no
	14	C/T	1b	no
	18	T/T	4c/4d	no
	21	C/T	3a	yes
	23	unknown	1b	yes
	25	C/C	undetermined	no
	28	C/C	4c/4d	no
	30	C/T	1b	no
	31	C/T	3a	no
	34	T/T	4c/4d	no
	39	C/T	3a	no
	40	T/T	4c/4d	no
	42	C/T	1b	no
	67	C/C	1b	no
	75	unknown	1b	yes
	89	C/C	3a	no
	91	C/C	4c/4d	yes
	92	T/T	3a	yes
	136	unknown	4c/4d	no
undetermined	47	unknown	4c/4d	no
no data^a^	1	C/C	4c/4d	yes
	174	C/C	undetermined	yes

^a^ In these two patients all follow up samples were HCV RNA negative preventing SSCP analysis

In the six patients in whom the SSCP band pattern was characterized as shifted, the relevant PCR products were cloned and sequenced. Only samples differing on SSCP analysis were thus analyzed, which means that when a number of samples from the same patient demonstrated identical SSCP band pattern, only one was cloned and sequenced. As seen in Fig 2, a novel viral genotype was identified in all but one of these patients. All six patients were infected with 3a strains at the beginning of therapy (in one case a mixed infection with genotypes 3a and 4 was identified), while the new genotypes were classified as type 4 (3 cases), type 1a (one case) and type 1b (one case). In one case (patient 27) the SSCP shift resulted from a single nucleotide substitution in the baseline 3a strain (Fig 2).

## Discussion

HCV heterogeneity in the natural course of infection as well as in the setting of antiviral therapy has been extensively studied. However, the majority of published studies on HCV quasispecies dynamics focused on highly variable regions of the virus, especially hypervariable region 1 (HVR1) [16, 25–31]. In the studies conducted by Farci et al. [25] and Nasu et al. [26], a clear reduction in genetic diversity leading to an increasingly homogeneous viral population was a consistent feature associated with therapy-induced viral clearance, while stability of quasispecies population favored viral persistence. In contrast, acute resolving hepatitis was reported to be associated with relative evolutionary stasis, whereas progressing acute hepatitis correlated with the presence of HCV genetic evolution [21, 27]. While studies on 5'UTR variability with respect to therapy outcome are rare [17, 32–35], it was reported that HCV quasispecies heterogeneity [17, 35] as well as mutations localization within IRES [32] may correlate with treatment outcome. However, others did not find any correlation [33, 34].

Relatively little is known about the association between HCV quasispecies and treatment outcome among HCV/HIV coinfected patients. A recent study by Sherman et al [16] demonstrated that, similar to monoinfected patients, an early HVR1 selection predisposes to SVR. Furthermore, both in HCV/HIV coinfection and HCV monoinfection, treatment outcome was related to HVR1 quasispecies complexity before treatment [16]. However, some other studies did not find such a correlation [28].

Our results showed that 5'UTR heterogeneity correlates with interferon and ribavirin treatment outcome. In the analyzed group of HCV/HIV coinfected patients, viral quasispecies stability during treatment favored viral persistence, whereas decrease in the number of variants and/or emergence of new variants was associated with SVR. These results are in agreement with those reported for HVR1 by Sherman in HCV/HIV coinfected patients [16], as well as with Farci's studies on HVR1 in HCV monoinfected patients [25]. Furthermore, our current observations are also compatible with our previous work on 5’UTR in HCV-positive/HIV-negative patients [17]. Thus, it seems that regardless of HIV status, both HVR1 and 5'UTR quasispecies behave similarly in the setting of antiviral treatment: their stability portends poor prognosis, while changes correlate with virus elimination. However, appearance of 'shift' in SSCP band pattern in HIV-positive and HIV-negative patients reflected two fundamentally different phenomena: among HCV monoinfected patients it represented mutations within the dominant infecting strain, while among HCV/HIV coinfected patients it reflected genotype change. Furthermore, among HCV monoinfected patients differences in SSCP band pattern between serum and PBMC at baseline were common and correlated with treatment outcome [17]. Surprisingly, in our group of HCV/HIV coinfected subjects no differences between these compartments were found. Thus, despite some similarities, the current study revealed significant differences in viral dynamics between HCV monoinfected and HCV/HIV coinfected patients receiving antiviral treatment.

Importantly, among 6 patients in the 'shift' group, in 3 patients the changes were confined to the PBMC compartment. Thus, without analyzing of PBMC compartment, 3 out of 6 shifts would have been missed and the correlation between this phenomenon and treatment outcome would not reach statistical significance.

HVR1 contains a potent epitope and changes within this region could be viewed as evidence of enhanced immune pressure driven by treatment. The important role of immune response in shaping HVR1/E2 region is demonstrated by the low diversity and complexity of quasispecies among immunosuppressed patients, including patients undergoing bone marrow transplantation [36], hypogammaglobulinemic patients [37], and HIV/HCV coinfected individuals with low CD4 T cell counts [38].

However, why interferon would affect selection of 5'UTR variants is less clear. The latter contains Internal Ribosomal Entry Site (IRES) and changes within this region are likely to affect translation, and by implication, replication efficiency. These could have direct clinical implications, as it was shown that in the immunocompetent host there may be selection of low translation efficiency HCV variants over the course of infection, while in immunosuppressed subjects the opposite could be true as low translation efficiency variants are superseded by high translation efficiency variants [39].

In 5 out of 6 patients in whom SSCP pattern suggested sequence changes, the new variants were found to belong to a different genotype altogether (Fig 2). Hypothetically, this could have been due to superinfection with a new virus, as such situation often results in rapid predominance of superinfecting strain, as described in the settings of liver transplantation and blood transfusion [12, 40]. While repeated exposure to HCV infection is common among IDU patients who constituted the study group, all patients remained abstinent for at least a year prior to the treatment and were in a methadone maintenance program. Another and far more likely possibility is that these patients were infected with at least two different genotypes, one of which was predominant at baseline but at the same time was more susceptible to treatment. Once replication of this strain has been halted, the minor strain belonging to a different genotype could become predominant and thus detectable. This scenario is given credence by the observation that in each of these cases, the initially dominant genotype was 3a, which is more sensitive to interferon/ribavirin therapy than either type 1a or type 4 genotypes.

In our study neither the IL-28B polymorphism nor viral genotype correlated with the treatment outcome, which is most likely due to the limited size of the study, as the expected trends were present, but did not reach statistical significance (Table 2). It is intriguing that the SSCP band pattern changes were more relevant with respect to SVR than either IL28B or genotypes.

In summary, we found correlation between HCV 5’UTR changes and treatment outcome in HCV/HIV coinfected patients. 5’UTR stability correlated with non-response, whereas changes in this region were associated with SVR. In addition, emergence of new HCV genotypes during treatment was common among our HCV/HIV coinfected IDU patients.
